# Evaluation of weed control efficacy and crop safety of the new HPPD-inhibiting herbicide-QYR301

**DOI:** 10.1038/s41598-018-26223-9

**Published:** 2018-05-21

**Authors:** Hengzhi Wang, Weitang Liu, Kongping Zhao, Hui Yu, Jia Zhang, Jinxin Wang

**Affiliations:** 1 College of Plant Protection, Shandong Agricultural University, Tai’an, 271018 China; 2 The Institute for the Control of Agrochemicals of Shandong Province, Ji’nan, 250013 China; 3 The Institute for the Control of Agrichemicals of Ministry of Agriculture, Beijing, 100125 China

## Abstract

QYR301, 1,3-Dimethyl-1H-pyrazole-4-carboxylic acid 4-[2-chloro-3-(3,5-dimethyl-pyrazol-1-ylmethyl)-4-methanesulfonyl-benzoyl]-2,5-dimethyl-2H-pyrazol-3-yl ester, is a novel HPPD-inhibiting herbicide and was evaluated to provide a reference for post-emergence (POST) application under greenhouse and field conditions. The crop safety (180 and 360 g active ingredient (a.i.) ha^−1^ treatments) experiment revealed that wheat, paddy, garlic and corn were the only four crops without injury at both examined herbicide rates. The weed control efficacy (60 and 120 g a.i. ha^−1^) experiment showed that QYR301 exhibited high efficacy against many weeds, especially weeds infesting paddy fields. Furthermore, it is interesting that both susceptible and multiple herbicide resistant *Echinochloa crus-galli* (L.) Beauv. and *Echinochloa phyllopogon* (Stapf) Koss, two notorious weed species in paddy field, remained susceptible to QYR301. Further crop tolerance results indicated that 20 tested paddy hybrids displayed different levels of tolerance to QYR301, with the japonica paddy hybrids having more tolerance than indica paddy hybrids under greenhouse conditions. Results obtained from field experiments showed that QYR301 POST at 135 to 180 g a.i. ha^−1^ was recommended to provide satisfactory full-season control of *E. crus-galli* and *Leptochloa chinensis* (L.) Nees and to maximize rice yields. These findings indicate that QYR301 possesses great potential for the management of weeds in paddy fields.

## Introduction

Weeds are among the crop pests and they reduce crop economic yield by 10 to 15% yearly, according to the Food and Agriculture Organization of the United Nations^1^. Meanwhile, weeds are also the hosts of various crop pests and pathogens^2^. Herbicides with different sites of action are by far the most effective weed control tools developed, killing 90 to 99% of the weeds targeted^3–5^. More than 95% of United States cotton, soybean, maize and sugar beet acres are treated with herbicides for weed control^6^. In China, herbicide use has grown rapidly in the past two decades, and is an indispensable part of efficient food production^7^. Modern herbicides have been widely used in the world, with significant contributions to the high crop yield and food quality over the past 50 years^8^. However, famers are overly reliant on herbicide technology in crop fields and many other situations because of the many advantages of herbicides, and it was inevitable that herbicide-resistant weed populations would evolve with herbicide selection persistently applied to huge weed populations over vast areas without diversity^9^. The evolution of weed herbicide resistance is a stark example of rapid evolution, with major negative consequences. Weeds resistant to herbicides, particularly in major field crops (wheat, rice, maize, soybean), are a widespread problem and a significant challenge for global food production^9,10^.

With the emergence of serious problems caused by different resistant weeds, herbicides with new modes of action are badly needed to manage herbicide-resistant weeds. However, no major new mode of action has been introduced to the market for about 20 years, since discovery efforts were significantly diminished owing to the appearance of glyphosate-resistant crops, company consolidations and the high-risk investment required. Another reason might be that the best molecular target sites used to develop herbicides have already been discovered^11^. Thus, the design and synthesis of new herbicides based on herbicide molecular target sites that have already been discovered has become the main method of researchers.

Now, resistances to acetyl-coenzyme A carboxylase (ACCase, EC.6.4.1.2) inhibitors, acetohydroxyacid synthase (ALS, EC2.2.1.6) inhibitors and photosystem II inhibitors (PS II, EC1.10.3.9) have been identified in 48, 159 and 105 weed species, respectively. However, resistance to hydroxyphenylpyruvate dioxygenase (HPPD, EC.1.13.11.27)-inhibiting herbicides has been only detected in 2 weed species world-wide^12^. In consideration of target-site-based herbicide resistance and cross-resistance, the newest generation of HPPD inhibitors is clearly the new hope to solve current weed problems^13^.

**Figure 1:**
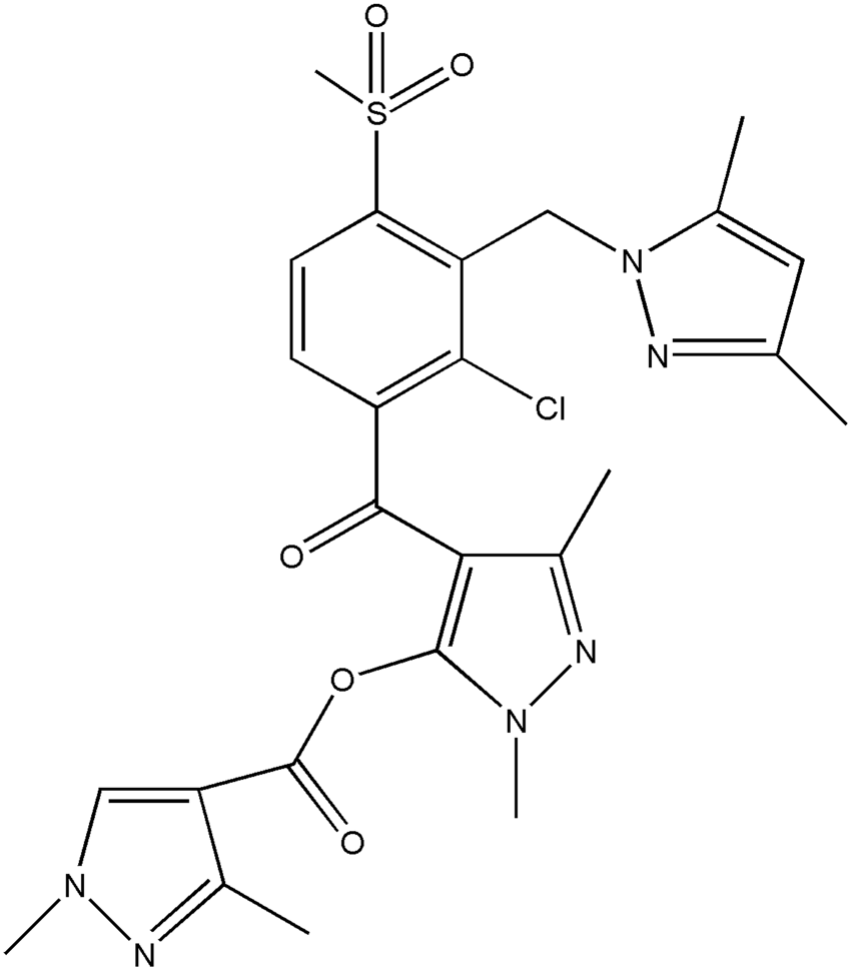
Structure of QYR301 used in the experiments, 1,3-Dimethyl-1*H*-pyrazole-4-carboxylic acid 4-[2-chloro-3-(3,5-dimethyl-pyrazol-1-ylmethyl)-4-methanesulfonyl-benzoyl]-2,5-dimethyl-2*H*-pyrazol-3-yl ester.

QYR301, 1,3-dimethyl-1H-pyrazole-4-carboxylic acid 4-[2-chloro-3-(3,5-dimethyl-pyrazol-1-ylmethyl)-4-methanesulfonyl-benzoyl]-2,5-dimethyl-2H-pyrazol-3-yl ester (Fig. 1), is a novel HPPD-inhibiting POST herbicide developed by Qingdao Kingagroot Chemicals Co., Ltd. and Shandong Agricultural University in 2011^14^. HPPD inhibitors consist of triketones, isoxazoles and pyrazoles. Triketones and isoxazoles are among the most widely used herbicides, such as mesotrione^15^ and isoxaflutole^16^. Topramezone, a relatively newer HPPD-inhibiting herbicide, was commercially introduced in 2006 and belongs to new chemical class of pyrazolones^17^. QYR301 also belongs to the chemical class of pyrazolones.

To date, QYR301 has been in registration process for use in China. Considering the diversity of crops and weeds in China, the objectives of this study were (1) to find an applicable crop by determining the efficacy of QYR301 against 31 common weeds and the crop safety for 22 crops in greenhouse conditions, (2) to determine the QYR301-tolerance of 20 safe crop hybrids in greenhouse conditions, (3) to identify the selectivity of QYR301 between a common Chinese safe crop hybrid and common weeds in greenhouse conditions, (4) to characterize the efficacy of QYR301 against resistant weeds in greenhouse conditions, and (5) to evaluate weed control, crop response and paddy yield in field conditions with QYR301 POST.

## Materials and Methods

### Herbicide formulation

QYR301 (Qingdao Kingagroot Chemicals Co., Ltd, Qingdao, China) with 97% purity was dissolved in a proper volume of acetone as a stock solution. The stock solution was diluted with 1% aqueous solution of Tween-80 (Solarbio Life Sciences, Beijing, China) to obtain the necessary rate for the crop safety, weed spectrum, paddy hybrid tolerance and selectivity index studies. The formulation of 6% QYR301 OD was kindly provided by Qingdao Kingagroot Chemicals Co., Ltd, Qingdao, China. QYR301 (6%OD) and penoxsulam (Daojie®, 25 g/L OD, Dow AgroSciences, Jiangsu, China) were dissolved and diluted with water to obtain the required application rates for the activity on resistant weeds in greenhouse and field experiments.

### Greenhouse experiment design

All the greenhouse experiments were conducted from October 2015 to September 2016 in a controlled greenhouse at Shandong Agricultural University, Tai’an, China (36.20°N, 117.13°E). The greenhouse conditions were maintained as follows: 12/12 h (light/dark) photoperiod achieved with natural light and augmented with supplemental lights (400-W high-pressure sodium lamp, 400 W, Philips, Amsterdam, the Netherlands), 1,400 µmol s^−1^ m^−2^ average photosynthetic photon flux density (PPFD) across replications for daytime hours, daytime temperature 23 to 29 °C, night temperature15 to 19 °C, and 75 ± 5% relative humidity. Before planting, crop and weed seeds were immersed in distilled water at 25 °C under a 12-h photoperiod in a growth chamber to accelerating germination (Model RXZ, Ningbojiangnan Instrument Factory, Ningbo, China). After radicals were visible, a proper number of germinated seeds were sown below the soil surface in plastic pots (16-cm -diameter and 13-cm -height) according to the size of crop seeds and seedlings. The soil, with a pH of 6.8 and 1.7% organic matter, was passed through a 3-mm sieve. After emergence, seedlings were thinned to a proper number of plants per pot. The pots were watered as needed to maintain moisture. Seedlings were treated with herbicides using a research track sprayer (Model ASS-4, National Agricultural Information Engineering and Technology Center of China) with a Teejet 9503EVS flat-fan nozzle that delivered a 450 L ha^−1^ spray of solution at a spray pressure of 275 kPa. All the experiments in the greenhouse were arranged in a randomized complete block design with three replicates and conducted twice.

#### Crop safety

Crops used in this study are presented in Table 1. These crops are conventional crops with relatively large planting area in China. Seedlings at the three- to five-leaf growth stage were treated with QYR301 at the rates of 180 and 360 g a.i. ha^−1^, and an untreated control was established for each crop species. Other experimental conditions were the same as described in the greenhouse experiment design. Herbicide damage of the seedlings was visually estimated by independent assessors 21 d after treatment (DAT) using a scale of 0 to 100% (0 = no damage, 100 = total death)^18^. Observation of plant whitening was also recorded to describe treatment performance.

**Table 1:** Visual injury ratings of trial crops treated with QYR301 POST application relative to the nontreated control in a greenhouse study 21 d after treatment (DAT).

Crops	Crop injury rating^a^ (SE)	
	180 g a.i. ha^−1^	360 g a.i. ha^−1^
	%	
Wheat (*Triticum aestivum* L.)	0	0 NS^b^
Paddy (*Oryza sativa subsp*. Keng)	0	0 NS
Garlic (*Allium sativum* L.)	0	0 NS
Corn (*Zea mays* L.)	0	0 NS
Cotton (*Gossypium hirsutum* L.)	17 (2.4)	56 (4.7)*
Mung bean [*Vigna radiata* (L.) Wilczek]	18 (3.0)	50 (5.6)*
Sorghum [*Sorghum bicolor* (L.) Moench]	20 (6.4)	32 (2.0)*
Peanut (*Arachis hypogaea* L.)	23 (2.1)	54 (2.5)*
Pepper (*Capsicum annuum* L.)	27 (3.7)	48 (3.4)*
Small red bean [*Vigna angularis* (Willd.) Ohwi et Ohashi]	32 (6.2)	73 (2.5)*
Watermelon [*Citrullus lanatus* (Thunb.) Matsum. et Nakai]	34 (0.9)	75 (1.2)*
Soybean [*Glycine max* (L.) Merr.]	35 (4.6)	89 (4.6)*
Potato (*Solanum tuberosum* L.)	38 (4.2)	70 (4.4)*
Cucumber (*Cucumis sativus* L.)	54 (2.1)	78 (4.1)*
Rape (*Brassica napus* L.)	62 (4.5)	94 (2.3)*
Tomato (*Lycopersicon esculentum* Mill.)	67 (5.0)	92 (3.4)*
Onion (*Allium fistulosum* L.)	71 (5.3)	94 (3.5)*
Eggplant (*Solanum melongena* L.)	75 (5.9)	88 (4.3)*
Coriander (*Coriandrum sativum* L.)	88 (2.1)	91 (5.4) NS
Sunflower (*Helianthus annuus* L.)	93 (1.8)	100*
Carrot (*Daucus carota* L. var. sativa Hoffm.)	93 (2.0)	100*
Radish (*Raphanus sativus* L.)	99 (0.7)	99 (0.9) NS

^a^Injury rating scale: 0 = consistent with control treatment, 0–30% = cotyledon and minority of functional leaves showed whitening, except newborn leaves, 30–60% = cotyledon, minority of functional leaves and new leaves showed whitening, 60–100% = majority of the plants showed serious whitening symptoms, some plant even necrosis, 100% = all plants showed whitening symptoms and necrosis. ^b^Significant differences between the two rates at the P < 0.05 level according to Fisher’s protected LSD test. *Significant; NS, not significant.

#### Weed spectrum

Weed species used in this study are presented in Table 2. These weed species are common weeds investing summer and winter crops fields in China. Seedlings were treated with QYR301 at the rates of 60 and 120 g a.i. ha^−1^ when they had reached the two- to four-leaf growth stage, and an untreated control was established for each weed species. Other experimental conditions were the same as described in the greenhouse experiment design. At 21 DAT, the shoots were harvested and oven dried at 72 °C for 72 h, and their dry weights were recorded.

**Table 2:** Dry weight inhibitions of trial weeds treated with QYR301 relative to the nontreated control in a greenhouse study 21 d after treatment (DAT).

Weeds	Dry weight inhibition (SE)	
	60 g a.i. ha^−1^	120 g a.i. ha^−1^
	%	
Shepherd’s purse [*Capsella bursa-pastoris* (L.) Medic.]	96 (1.0)	96 (1.8) NS^a^
Rice flat sedge [*Cyperus iria* L.]	94 (2.2)	95 (1.2) NS
Barnyard grass [*Echinochloa crus-galli* (L.) Beauv.]	83 (1.7)	87 (1.3)*
Black nightshade [*Solanum nigrum* L.]	82 (1.3)	98 (1.1)*
Small goosefoot [*Chenopodium serotinum* L.]	81 (2.8)	94 (1.1)*
False daisy [*Eclipta prostrata* L.]	81 (2.6)	95 (0.5)*
Sheathed monochoria herb [Monochoria vaginalis (Burm. F.) Presl ex Kunth]	79 (2.3)	91 (0.8)*
Crickweed [*Myosoton aquaticum* (L.) Moench]	60 (3.3)	68 (3.6)*
Goosegrass [*Eleusine indica* (L.) Gaertn.]	67 (3.3)	85 (0.2)*
Chinese sprangletop [*Leptochloa chinensis*(L.) Nees]	67 (3.0)	93 (0.4)*
Late watergrass [*Echinochloa phyllopogon* (Stapf) Koss.]	46 (2.2)	79 (1.5)*
Chinese green foxtail [*Setaria viridis* (L.) Beauv.]	44 (4.5)	57 (4.0)*
Asia minor bluegrass (*Polypogon fugax* Nees ex Steud.)	42 (5.4)	65 (3.9)*
Herba portulacae (*Portulaca oleracea* L.)	40 (4.5)	46 (1.9)*
Herb of spanishneedles (*Bidens pilosa* L.)	36 (4.7)	50 (4.7)*
Ipomoea purpurea [*Pharbitis purpurea* (L.) Voigt]	35 (2.9)	46 (4.5)*
Carolina cranesbill herb (*Geranium carolinianum* L.)	31 (2.9)	30 (4.5) NS
Velvetleaf (*Abutilon theophrasti* Medicus)	22 (1.4)	43 (2.1)*
Purple nutsedge (*Cyperus rotundus* L.)	16 (1.0)	19 (4.5) NS
Rushlike bulrush (*Scirpus juncoides* Roxb.)	15 (4.4)	31 (3.7)*
Siberian cocklebur (*Xanthium sibiricum* Patrin ex Widder)	14 (4.5)	34 (4.2)*
Wild oat (*Avena fatua* L.)	6 (4.0)	11 (2.0)*
Japanese foxtail (*Alopecurus japonicus* Steud.)	—^b^	8 (2.4)*
Eleocharis yokoscensis [*Heleocharis yokoscensis* (Franch. et Savat.) Tang et Wang]	—	—NS
Catchweed (*Galium aparine* L.)	—	—NS
Blugrass (*Poa annua* L.)	—	—NS
Digitaria sanguinalis [*Digitaria sanguinalis* (L.) Scop.]	—	39 (6.2)*
Water foxtail (*Alopecurus aequalis* Sobol.)	—	23 (2.1)*
Keng Stiffgrass [*Sclerochloa kengiana* (Ohwi) Tzvel.]	—	9 (3.0)*
Redroot pigweed (*Amaranthus retroflexus* L.)	—	62 (0.9)*
American sloughgrass [*Beckmannia syzigachne* (Steud.) Fern.]	—	—NS

^a^Significant differences between the two rates at the P < 0.05 level according to Fisher’s protected LSD test. *Significant; NS, not significant. ^b^Dry weight inhibition ≤5%.

#### Paddy hybrid tolerance

Considering the high crop safety of QYR301 for the tested paddy and its high efficacy against some weeds infesting paddy fields, QYR301 was recommended to be applied to paddy fields. The tolerances of more paddy hybrids were tested here. The paddy hybrids used in this study are on sale in Chinese agricultural market and presented in Table 3. Six seeds were sown per pot. Seedlings at the three- to five-leaf growth stage were treated with QYR301 at the rates of 180 and 360 g a.i. ha^−1^ and an untreated control was established for each paddy hybrid. Other details of the experimental design were the same as in the crop safety section.

**Table 3:** Visual injury ratings of trial paddy hybrids treated with QYR301 POST application relative to the nontreated control in a greenhouse study 21 d after treatment (DAT).

Indica	Paddy injury rating^a^ (SE)		Japonica	Paddy injury rating^a^(SE)	
	180 g a.i.ha^−1^	360 g a.i.ha^−1^		180 g a.i.ha^−1^	360 g a.i.ha^−1^
	%			%	
Liangyouzao17	24 (3)	58 (9)*^b^	Yujing6	0	5 (3)*
Liangyou287	11 (4)	53 (4)*	Huajing5	0	0 NS
Ganyuan100	0	19 (3)*	Huaidao5	0	0 NS
Fengliangyou1	0	15 (2)*	Lianjing11	0	0 NS
Rongyouhuazhan	0	15 (2)*	Longjing46	0	0 NS
Neixiang10	0	11 (2)*	Ningjing28	0	0 NS
Yliangyou1	0	5 (1)*	Yanfeng47	0	0 NS
Zhongjiazao17	0	7 (2)*	Zhendao99	0	0 NS
Jinxiangnuo	0	0 NS	Fuyuan4	0	0 NS
Huanghuazhan	0	0 NS	Longjing47	0	0 NS

^a^Injury rating scale: 0 = consistent with control treatment, 0–30% = cotyledon and minority of functional leaves showed whitening, except new leaves, 30–60% = cotyledon, minority of functional leaves and newborn leaves showed whitening, 60–100% = majority of the plants showed serious whitening symptoms, some plant even necrosis, 100% = all plants showed whitening symptoms and necrosis. ^b^Significant differences between the two rates P < 0.05 level according to Fisher’s protected LSD test. *Significant; NS, not significant.

#### Selectivity index (SI)

The ratio of the rate that caused a 10% crop growth reduction and the rate that caused a 90% weed growth reduction is the selectivity index^19^. Ningjing 28 (NJ 28)and Zhongjizao 17 (ZJZ 17) are two main cultivated paddy hybrids in the Yellow River irrigation area and at the middle and lower reaches of the Yangtze River, respectively, in China. *E. phyllopogon*, *Capsella bursa-pastoris* (L.) Medic., *Cyperus iria* L., *E. crus-galli*, *Solanum nigrum* L., *Eclipta prostrata* L., *Monochoria vaginalis* (Burm. F.) Presl ex Kunth, and *L. chinensis* are the common weed species in Chinese paddy production systems. To quantify the SIs between tolerant paddies and weed species under POST application, NJ 28 was treated with 0, 540, 1080, 1440, 2160, 4320, and 8640 g a.i. ha^−1^; ZJZ 17 was treated with 0, 360, 540, 720, 1080, 1440, and 2160 g a.i. ha^−1^; *E. phyllopogon* was treated with 0, 11.25, 22.5, 45, 90, 180, and 360 g a.i. ha^−1^; *E. prostrata*, *L. chinensis*, *C. iria*, *M. vaginalis* and *E. crus-galli* were treated with 0, 3.75, 7.5, 15, 30, 60, and 120 g a.i. ha^−1^; *C. bursa-pastoris* was treated with 0, 1.875, 3.75, 7.5, 15, 30, and 60 g a.i. ha^−1^, and *S. nigrum* was treated with 0, 0.1172, 0.4686, 1.875, 7.5, 30, and 120 g a.i. ha^−1^. The treatments of the paddies and the weeds with the herbicide were conducted simultaneous under the same conditions. Other experimental conditions were the same as described for the greenhouse experiment design. At 21 DAT, the shoots were harvested and oven dried at 72 °C for 72 h, and the dry weights were recorded.

#### Activity of QYR301 against resistant weeds in paddy

The activity of QYR301 against two herbicide-resistant *Echinochloa* Beauv. species, *E. phyllopogon* and *E. crus-galli*, which are wide spread in paddy fields, was also characterized in this study. The herbicides doses applied to the susceptible (S) and resistant (R) biotypes of *E. phyllopogon* and *E. crus-galli*, known to be resistant from previous testing, are listed in Table 4. Other experimental design and conditions were the same as in the weed spectrum section.

**Table 4:** Dry weight inhibitions of ALS- and ACC-resistant *E. phyllopogon* and *E. crus-galli* treated with QYR301 relative to the nontreated control in a greenhouse study 21 d after treatment (DAT).

Treatments (g a.i.ha^−1^)	Dry weight inhibition (SE)					
	*E. phyllopogon* S^a^	*E. phyllopogon* ALS-R^a^	*E. phyllopogon* ACC-R^a^	*E. crus-galli* S	*E. crus-galli* QR^a^	*E. crus-galli* ALS-R
	%					
Penoxsulam (15)	70 (1.7) de	0 g	—	92 (2.2) a	—	0 c
Cyhalofop-butyl (80)	84 (4.5) b	—	8 (3.9) f	—	—	—
Quinclorac (250)	—	—	—	92 (1.3) a	32 (4.3) b	—
QYR301 (60)	—	—	—	91 (1.1) a	91 (2.2) a	91 (1.5) a
QYR301 (120)	78 (4.6) c	68 (3.4) e	74 (2.7) cd	92 (2.4) a	92 (1.2) a	90 (2.5) a
QYR301 (180)	95 (1.1) a	93 (1.0) a	94 (0.8) a	—	—	—

^a^S = susceptible, ALS-R = ALS-resistant, ACC-R = ACC-resistant, QR = quinclorac-resistant. Different letters indicate significant difference at the P < 0.05 level according to Fisher’s protected LSD test.

### Field experiment

Field experiments were conducted twice during the paddy growing seasons in 2017 at the Research Farm of Shandong Agricultural University (35.53°N, 116.52°E, altitude 40 m, yearly average precipitation of 631.6 mm, loam with 1.6% organic matter and a pH of 7.1) in Ji’ning (JN) and farmer fields (38.74°N, 106.47°E, altitude 1101 m, yearly average precipitation of 180.1 mm, loam with 1.4% organic matter and a pH of 8.3) in Shi’zui’shan (SZS), China. The test fields at both JN and SZS were heavily infested with *E. crus-galli* and *L. chinensis*, and penoxsulam had failed to control the *E. crus-galli* at the SZS location^20^. Tiyou267 paddy (japonica) was direct-seeded in the test fields at JN on June 5, 2017 and Ningjing48 (japonica) was direct-seeded in the test fields at SZS on April 19, 2017. The weed density in JN and SZS was 72 to 80 and 80 to 90 plants m^−2^ for *E. crus-galli*, 30 to 35 and 20 to 30 plants m^−2^ for *L. chinensis*, respectively. The monthly air temperature and precipitation at the experimental site are presented in Table 5.

**Table 5:** Monthly air temperature and total precipitation at the experimental sites at Ji’ning (JN) and Shi’zui’shan (SZS) during the paddy growing season in 2017.

Month	Air temperature (°C)						Total precipitation (mm)	
	Maximum		Minimum		Mean			
	JN	SZS	JN	SZS	JN	SZS	JN	SZS
May	36.5	34.9	0	0	23.0	18.4	58.4	15.1
June	36.2	34.5	16.1	0	24.9	22.5	62.4	24.1
July	36.7	40.1	22.3	14.5	28.1	25.6	271.3	40.2
August	36.1	35.6	22.5	11.6	26.6	22.0	82.6	40.1
September	31.8	30.3	16.5	3.3	22.4	18.8	11.5	23.6

Experiments were designed as randomized complete blocks with 4 replications with plot areas of 20 m^2^ (5 m × 4 m). The treatments included four rates of 90, 135, 180, and 270 g a.i. ha^−1^, a single field recommended rate of 30 g a.i. ha^−1^ penoxsulam, a hand weed control (using a hand hoe at 0, 20, 40 DAT), and a weedy control, resulting in a total of 7 treatments as shown in Table 6. In wet-seeded rice, water was partially drained from fields, and the POST application was made on June 23, 2017 at JN (cloudy, 18 to 29 °C, light air) and May 10, 2017 at SZS (cloudy, 8 to 27 °C, gentle breeze) when paddy were at the two leaves and one half leaf period. A total spray volume of 450 L/ha was applied with a backpack sprayer (Bellspray Inc., Opelousa, LA) equipped with a single 8002 VS nozzle (Teejet Technologies, Wheaton, IL), and paddies were flooded within 48 hours after application. Rice production systems followed the local common farming practice.

**Table 6:** Visual estimates of percent weed control following different herbicide treatments in 2017 at Ji’ning (JN) and Shi’zui’shan (SZS).

Treatments (g a.i. ha^−1^)	Percent weed control^a,b^							
	*E. crus-galli*				*L. chinensis*			
	20DAT		40DAT		20DAT		40DAT	
	JN	SZS	JN	SZS	JN	SZS	JN	SZS
	%				%			
QYR301 (90)	90.0 b	88.0 d	84.3 c	83.8 c	54.8 d	50.8 d	47.3 d	47.0 d
QYR301 (135)	94.5 b	93.8 c	92.8 b	92.8 b	88.5 c	86.0 c	85.8 c	84.0 c
QYR301 (180)	97.8 a	96.3 b	96.0 a	96.8 a	95.0 b	93.5 b	93.8 b	92.5 b
QYR301 (270)	98.0 a	98.8 a	97.5 a	97.8 a	98.3 a	98.0 a	97.0 a	96.8 a
Penoxsulam (30)	97.5 a	33.8 e	96.3 a	32.8 d	34.5 e	25.3 e	30.5 e	24.8 e
Hand weeding	—	—	—	—	—	—	—	—
Weedy control	—	—	—	—	—	—	—	—

Visual estimates of percent weed control following different herbicide treatments in 2017 at Ji’ning (JN) and Shi’zui’shan (SZS). ^a^Visual estimates of percent weed control were recorded 20 and 40 d after treatment (DAT) using a scale of 0 to 100% where 0 = no weed control and 100 = complete weed control. ^b^Different letters are significantly different at the P < 0.05 level according to Fisher’s protected LSD test.

Visual estimates of percent weed control were recorded 20 and 40 DAT using a scale of 0 to 100%, where 0 = no weed control and 100 = complete control. The visual crop injury was evaluated at 3, 5, 10 and 20 DAT using a scale of 0 to 100%, where 0 = no crop injury and 100 = plant death^21^. Rice yields were harvested from in 5 m^2^ in each plot and, weighed, and then rice yield was expressed on a per hectare basis.

### Statistical analysis

The data from the repeated experiments in the greenhouse were analyzed by analysis of variance (ANOVA) (Version 17.0; IBM Corporation, Armonk, NY). Data were pooled because of the nonsignificant interaction with two repeated experiments, and the means were separated using Fisher’s protected least-significant difference (LSD) at the P < 0.05 significance level.

All regression analyses were conducted using SigmaPlot software (Version 13.0; Systat Software Inc., CA, USA). The rate-response curves were obtained by non-linear regression using the four-parameter log-logistic response equation 1^22^:

1 $$y=c+(d-c)/\{1+\exp [b(\mathrm{log}\,x-\,\mathrm{log}\,G{R}_{50})]\}$$

In this model, *b* is the relative slope of the herbicide dose resulting in 50% growth inhibition (*GR*_50_), *c* is the lower limit and *d* is the upper limit. The herbicide dose was the independent variable (*x*), and the growth response (percentage of the untreated control) was the dependent variable (*y*) in the regression equation.

Based on the regression parameters, the *GR*_10_, *GR*_50_ and *GR*_90_ values for herbicide selectivity were calculated^23^. The *SIs* of QYR301 were calculated according to equation 2:

2 $$S{I}_{(10,90)}=G{R}_{10(crop)}/G{R}_{90(weed)}$$

where *GR*_10_ is the herbicide dose causing 10% growth inhibition of paddy and *GR*_90_ is the herbicide dose causing 90% growth inhibition of the trial weeds.

The data from the field experiments were subjected to ANOVA, and the means were separated using Fisher’s protected LSD test at the P < 0.05 significance level.

### Data availability

All data generated or analysed during this study are included in its Supplementary Information files.

## Results

### Crop safety

Among all the trial crops, wheat, paddy, garlic and corn were the four crops that were not injured, even when treated with QYR301 at the rate of 360 g a.i. ha^−1^ (Table 1). However, sunflower, coriander, carrot and radish were seriously necrotic when treated at the rate of 180 g a.i. ha^−1^ (Table 1).

### Weed spectrum

At the rate of 60 g a.i. ha^−1^, the application of QYR301 had a high efficacy (dry weight inhibition >80%) against 6 of the weeds tested in this experiment, including *C. bursa-pastoris*, *C. iria*, *E. crus-galli*, *S. nigrum*, *Chenopodium serotinum* L. and *E. prostrata*, and the number increased to 9 when treated with QYR301 at the rate of 120 g a.i. ha^−1^ (Table 2). However, some weed species were not susceptible (dry weight inhibition <20%) to QYR301 even when treated at the rate of 120 g a.i. ha^−1^, including *Avena fatua* L., *Alopecurus japonicus* Steud., *Heleocharis yokoscensis* (Franch. et Savat.) Tang et Wang, *Galium aparine* L., *Poa annua* L., *Sclerochloa kengiana* (Ohwi) Tzvel. and *Beckmannia syzigachne* (Steud.) Fern.

### Paddy hybrid tolerance

In the weed spectrum study, the weeds QYR301 have high efficacy against mainly infested the paddy fields. Therefore, a paddy hybrid tolerance experiment was also conducted to further evaluate for the application of this herbicide in paddy fields. QYR301 was found to be safe for most of the 20 tested paddy hybrids under POST application at 180 g a.i. ha^−1^ in the greenhouse, except Liangyouzao 17 and Liangyou 287, whose herbicide damage scores were 24% and 11%, respectively (Table 3). However, there were obvious differences on sensitivity to QYR301 between indica and japonica when the paddy hybrids were treated POST at 360 g a.i. ha^−1^. For example, no injury was observed for the japonica paddy hybrids, except Yujing6 with only 5% herbicide damage (some japonica hybrid showed whitening at 3 DAT, but all hybrids recovered at about 7 DAT), whereas almost all 10 indica paddy hybrids, especially Liangyouzao17 and Liangyou287 whose injury ratings were at least 53%, were sensitive to QYR301 (Table 3).

### Selectivity index

**Figure 2:**
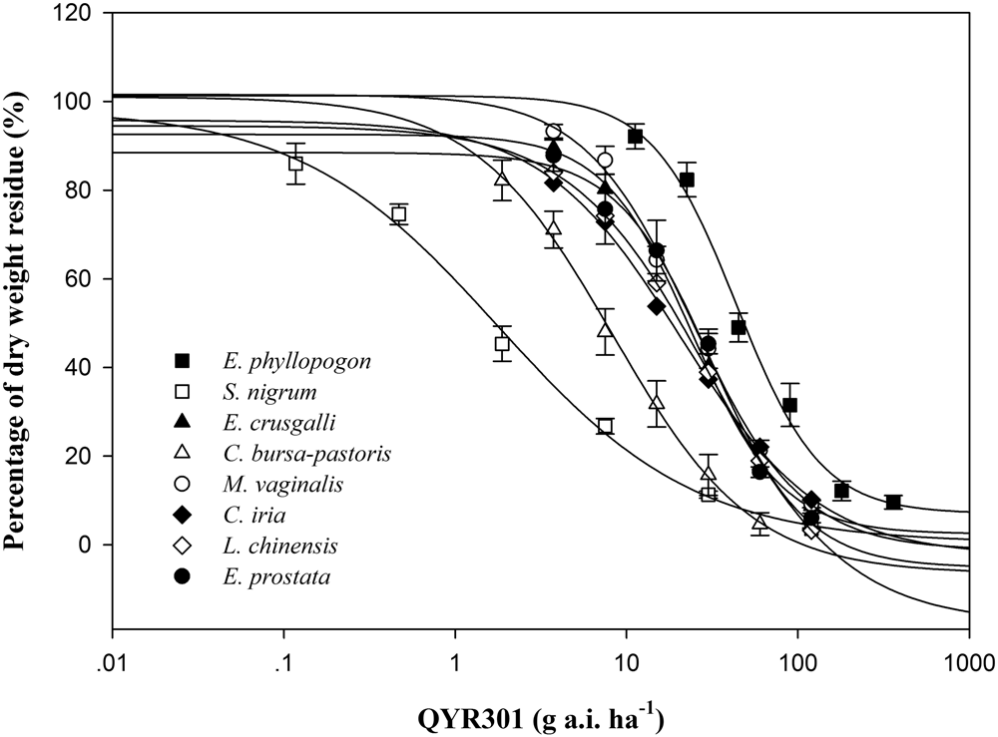
Percentage of dry weight residue of *E. phyllopogon*, *S. nigrum*, *E. crus-galli*, *C. bursa-pastoris*, *M. vaginalis*, *C. iria*, *L. chinensis* and *E. prostrata* response to increasing QYR301 rates in greenhouse study 21 day after treatment (DAT) based on nonlinear regression fit to a four parameter log–logistic curve model; *y* = *c* + (*d* − *c*)/{1 + exp[*b*(log*x* − log*GR*_50_)]}.

According to the results of the paddy hybrid tolerance experiment in the greenhouse, a dose-response study was conducted to confirm the SIs between two paddy hybrids, NJ 28 (japonica) and ZJZ 17 (indica), and eight weed species. Compared with the other eight tested weeds, *S. nigrum* and *C. bursa-pastoris* were more sensitive to QYR301(Fig. 2). *E. phyllopogon* was more tolerant than any other weed in the experiment (Fig. 2). QYR301 was safe for NJ28 and ZJZ17 when applied POST, with SI values of 3.1 to 30.5 (Table 7).

**Table 7:** Rates of QYR301 causing 10% and 50% growth reduction of Ningjing 28 (NJ 28) and Zhongjiazao 17 (ZJZ17), and 50% and 90% growth reduction of 8 weeds, and the selectivity index (SI) between 2 paddy hybrids and 8 weeds in a greenhouse study 21 d after treatment (DAT).

Trial plants	GR^a^_10_(SE)	GR_50_(SE)	GR_90_(SE)	SI^b^ NJ28	SI ZJZ17
	g a.i. ha^−1^				
NJ 28 (Japonica)	1089.4 (22.9)	1899.4 (26.8)	—	—	—
ZJZ 17 (Indica)	635.4 (19.2)	1116.5 (25.3)	—	—	—
*S. nigrum*	—	1.8 (0.5)	35.7 (6.8)	30.5	17.8
*C. bursa-pastoris*	—	8.4 (1.6)	41.1 (4.2)	26.5	15.5
*L. chinensis*	—	30.6 (0.3)	82.8 (1.2)	13.2	7.7
*E. prostrata*	—	31.1 (6.1)	85.4 (12.1)	12.8	7.4
*E. crus-galli*	—	24.3 (1.1)	93.6 (3.1)	11.6	6.8
*M. vaginalis*	—	24.3 (2.9)	107.6 (8.6)	10.1	5.9
*C. iria*	—	20.8 (2.7)	119.5 (10.1)	9.1	5.3
*E. phyllopogon*	—	43.8 (7.9)	205.6 (12.7)	5.3	3.1

^a^GR, growth reduction. ^b^SI, selectivity index. Selectivity index was calculated by SI (10,90) = GR_10(paddy)_/GR_90(weed)_.

### Activity of QYR301 against resistant weeds in paddy

QYR301 treatments had equal efficiency against the S, ALS-R and ACC-R biotypes of *E. phyllopogon* (>93% at 180 g a.i. ha^−1^, respectively), whereas penoxsulam had failed to control ALS-R biotypes (0% dry weight inhibition at 15 g a.i. ha^−1^) and cyhalofop-butyl had failed to control ACC-R biotypes with 8% dry weight inhibition at 80 g a.i. ha^−1^ (Table 4). Similarly, treatments with QYR301 are equally effective on the S, QR and ALS-R biotypes of *E. crus-galli* (all 91% dry weight inhibition at 180 g a.i. ha^−1^), whereas, quinclorac and penoxsulam had failed to control the QR biotypes with 32% and 0% dry weight inhibition, respectively (Table 4).

### Field experiment

Significant differences among visual estimates of percent weed control for herbicide treatments were observed in both JN and SZS (Table 6), and the data were consistent with the results of the greenhouse study to some extent. All weed species were more than 84% controlled by QYR301 at all tested rates increasing from 90 to 270 g a.i. ha^−1^, except for *L. chinensis*, which was only approximately 50% controlled by QYR301 at 90 g a.i. ha^−1^. QYR301 treatments controlled *E. crus-galli* with efficacy greater than 80% at the Shi’zui’shan location, where penoxsulam has failed to control *E. crus-galli* (Table 6). Compared to QYR301 at 20 DAT, a lower efficacy of less than 85% for *E. crus-galli* was observed with QYR301 application at 90 g a.i. ha^−1^ at 40 DAT, whereas a higher efficacy of over 92% was observed at 135, 180, 270 g a.i. ha^−1^ at 40 DAT (Table 6). It is worth mentioning that preemergence control for weeds was observed in treatments with application of QYR301.

Bleaching of newly developed tissue occurred when paddy QYR301 was applied at 270 g a.i. ha^−1^ to paddy fields at the JN location, however, it did not exceed 6% even when the most serious injury occurred at 5 DAT, and the plants recovered completely at 20 DAT (Table 8).

**Table 8:** Visual estimates of injury to rice and rice yields following different herbicide treatments at Ji’ning (JN) and Shi’zui’shan (SZS) in 2017.

Treatments (g a.i. ha^−1^)	Crop injury^a,b^								Rice yield (SE)^b^		Yield growth rate (SE)^b^	
	3DAT		5DAT		10DAT		20DAT		JN	SZS	JN	SZS
	JN	SZS	JN	SZS	JN	SZS	JN	SZS				
	%								kg ha^−1^		%	
QYR301 (90)	0 b	0	0 b	0	0 b	0	0	0	4290 (236) d	10830 (124) d	6.3 (5.9) c	5.9 (1.2) d
QYR301 (135)	0 b	0	0 b	0	0 b	0	0	0	4575 (111) bc	11160 (218) c	13.4 (2.8) b	9.1 (2.1) c
QYR301 (180)	0 b	0	0 b	0	0 b	0	0	0	4710 (76) ab	11820 (154) b	16.7 (1.9) ab	15.5 (1.5) b
QYR301 (270)	4.0 a	0	5.8 a	0	1.5 a	0	0	0	4770 (127) a	11925 (235) ab	18.2 (3.1) ab	16.6 (2.3) ab
Penoxsulam (30)	0 b	0	0 b	0	0 b	0	0	0	4525 (82) c	10260 (201) e	12.1 (2.0) b	0.3 (2.0) e
Hand weeding	—	—	—	—	—	—	—	—	4855 (143) e	12155 (164) e	20.3 (2.6) a	18.8 (1.2) a
Weedy control	—	—	—	—	—	—	—	—	4035 (104) a	10230 (119) a	—	—

^a^Visual crop injury was evaluated at 3, 5, 10, and 20 d after treatment (DAT) with the scale of 0 to 100%, where 0% represents no injury and 100% represents plant death. ^b^Different letters are significantly different at the P < 0.05 level according to Fisher’s protected LSD test.

Compared with the weedy control without herbicide application, the rice grain yields increased as the QYR301 rate increased from 90 to 270 g a.i. ha^−1^ (Table 8). The QYR301 rate at 270 g a.i. ha^−1^ resulted in 18.2% and 16.6% yield increases at the JN and SZS locations, respectively (Table 8). However, there was no significant difference in rice yield growth rate between treatments with QYR301 application at 180 g a.i. ha^−1^ and 270 g a.i. ha^−1^ (Table 8). Compared to the hand weeding plots, the weeds in the uncontrolled treatment and the penoxsulam treatment (failed to control) reduced yields by more than 18% (Table 8).

## Discussion

QYR301 is a novel HPPD-inhibiting herbicide, belonging to the new chemical class of pyrazolones. Pyrazolone derivatives have been synthesized for anticancer, antimicrobial, fungicidal and herbicidal activities, and many of them are effective against human pathogens^24–26^. Topramezone is the first herbicide that belongs to the chemical class of pyrazolones, and it is a selective POST herbicide used in maize crops to control annual grass and broad leaf weeds^19^. Topramezone is an HPPD inhibitor and prevents the biosynthesis of carotenoid that protects chlorophyll molecules from dangerous UV rays and excess light. There is nothing to prevent sunlight from penetrating into the leaves, which results in the photooxidation of chlorophyll molecules. Consequently, the weed turns white and dies^19^. QYR301 presents typical HPPD inhibitor activity^17^, and is a novel member of the HPPD inhibitors of homogentisic acid (HGA) biosynthesis.

According to crop safety experiments, QYR301 has potential to be used in wheat, paddy, garlic and corn fields. Tolerance by cotton, mung been, sorghum, peanut and pepper of QYR301 at 180 g a.i. ha^−1^ was also observed with new leaves consistent with the control treatment at 21 DAT. However, QYR301 was not safe for many crops tested in this experiment, especially sunflower, carrot and radish. Considering the herbicide residue, QYR301 is not recommended when paddy is in rotation with those crops.

Based on our weed spectrum experiments, QYR301 can effectively control 10 weed species (dry weight inhibition >75% at the rate of 120 g a.i. ha^−1^), including *C. bursa-pastoris*, *C. iria*, *E. crus-galli*, *S. nigrum*, *C. serotinum*, *E. prostrata*, *M. vaginalis*, *Eleusine indica* (L.) Gaertn., *L. chinensis* and *E. phyllopogon*. Although there are large differences between greenhouse and field conditions, the data obtained in the idealized greenhouse environment could provide application guidance in the field and reflect the real efficacy to some extent. For example, compared to *L. chinensis*, *E. crus-galli* was more sensitive to QYR301 in the greenhouse study. In accord with the greenhouse results, *E. crus-galli* was controlled effectively in the field with the application of QYR301at 90 g a.i. ha^−1^ (weed control >88%), however, QYR301 had a low efficacy against *L. chinensis* in field environmental conditions in this study (weed control <55%).

Eight of the ten weed species controlled by QYR301 are dominant weeds infesting paddy fields, such as *C. bursa-pastoris*, *C. iria*, *E. crus-galli*, *C. bursa-pastoris*, *L. chinensis*, *E. prostrata* and *E. phyllopogon*. Although QYR301 had low efficacy against some weeds infesting paddy fields, such as *Scirpus juncoides* Roxb. and *H. yokoscensis*, it could be mixed with other herbicides for a broader spectrum of weed control. Besides, paddy was not injured when treated with QYR301 at the tested application rates in the crop safety experiments. Based on these findings, QYR301 is recommended for application in paddy fields. Further study of the tolerance of paddy hybrids showed that indica paddy hybrids were more sensitive to QYR301 compared with japonica paddy hybrids, and it indicating that QYR301 should not exceed the recommended rates (135–180 g a.i. ha^−1^) when used in indica paddy fields. Here, the SI values for NJ28 (japonica), ZJZ17 (indica) and eight common weeds infesting paddy fields were also identified. The more the SI increases over 1.0, the more selective the herbicide is between the crop and the weeds^27^. According to the report by Bartley, an herbicide can be safely used on a crop when the SI value is greater than 2.0^28^. Thus, QYR301 was safe for NJ28 and ZJZ17 while effective against *S. nigrum*, *C. bursa-pastoris*, *L. chinensis*, *E. prostrata*, *E. crus-galli*, *M. vaginalis*, *C. iria* and *E. phyllopogon* when applied POST with SI values ranging from 3.1 to 30.5. Nevertheless, the present study had only evaluated the safety for 20 paddy hybrids. Paddy hybrids in China are numerous and have a wide and complex distribution in different regions. The sensitivity of more paddy hybrids to QYR301 will be evaluated to define the application range of rice hybrids in further experiments.

Especially, we found that QYR301 exhibits high efficacy against *E. crus-galli*, one of the worst weeds infesting paddy fields throughout the world due to the difficulty of controlling it^29^. The emergence of *E. crus-galli* as a problematic weed is due to its prolific seed production, its competitiveness and its use of the C_4_ photosynthetic pathway^30,31^. *E. crus-galli* can reduce rice yield by 30% and even cause crop failure if left uncontrolled. Many herbicides have been intensively used in most countries, which has resulted in the rapid evolution of herbicide resistance in *E. crus-galli*^32,33^. Although *E. crus-galli* and *E. phyllopogon* have been reported to be resistant to propanil^34^, quinclorac^35^, ALS inhibitors^36,37^, and/or ACCase inhibitors^38,39^, both were very susceptible to QYR301. We also observed that QYR301 had great efficiency against the S, ALS-R, ACC-R and quinclorac-R biotypes of *E. phyllopogon* and *E. crus-galli*. However, more attention should be paid to prevent or delay the occurrence of resistance to QYR301 and other HPPD inhibitors, especially in *E. crus-galli*. It is essential to reduce the herbicide selection to prevent or delay the occurrence of resistance by increasing crop diversity, natural biological controls, alternating between PRE and POST herbicide application, and rotating herbicide sites-of-action and mixtures^40^.

QYR301 had good efficiency against *L. chinensis* with POST application at a rate of 135 g a.i. ha^−1^, although it failed to control *L. chinensis* at rate of 90 g a.i. ha^−1^, which was consistent with the results of the greenhouse study. Meanwhile, the efficiency of QYR301 at rate of 90 g a.i. ha^−1^ against *E. crus-galli* had a relatively lager decline at 40 DAT compared to 20 DAT, and there may be a need for a second application in later field management. As seen in the greenhouse study, QYR301 had equally satisfactory efficacy against susceptible and penoxsulam-resistant biotypes of *E. crus-galli*. Slight injury to the paddy occurred at the JN location when the paddy was treated with QYR301 at a rate of 270 g a.i. ha^−1^. Several instances of HPPD inhibitor phytotoxicity have been reported, such as high use rate^41^, application time^42^ and varied susceptibility of crop hybrids to herbicides^43^. Some environmental factors such as wet and cold conditions can also result in crop injury by HPPD inhibitors^44^. The slight and temporary injury to the Tiyou267 paddy observed in this study may be related to the susceptibility of the paddy hybrid itself. More paddy hybrids should be tested in further research to define the lowest rate at which injury to different paddy hybrids occurs. All the tested QYR301 rates increased the rice yields; however, the paddy treated with QYR301 at 270 g a.i. ha^−1^ produced yields similar to those of the paddy treated with QYR301 at 180 g a.i. ha^−1^. All the facts taken together suggest that 135 to 180 g a.i. ha^−1^ of QYR301 is recommended according to our study.

Although hand weeding has the highest rice yields among seven treatments, it is economically unjustifiable because of the labour requirement and cost^45,46^, and a combination of hand weeding and chemical control may result in economical and efficient weed control in paddy fields.

In conclusion, results obtained from both the greenhouse and field experiments revealed that QYR301 has good potential for weed control in paddy production system as a selective POST herbicide. Benzobicyclon and mesotrione cause significant damage to paddy depending on hybrids^47,48^ and weeds are rapidly evolving resistance to multiple herbicides with various modes of action, such as propanil, quinclorac, ALS inhibitors, ACCase inhibitors and PSII inhibitors. With those challenges, QYR301, a novel HPPD-inhibiting herbicide, may be an effective and safe chemical agent to manage the herbicide resistance of some weed species in paddy fields.

## Electronic supplementary material

Supplementary Information
