# Supplementary material for: Evaluation of weed control efficacy and crop safety of the new HPPD-inhibiting herbicide-QYR301
Source: Sci Rep. 2018 May 21;8:7910. doi: 10.1038/s41598-018-26223-9 (PMC5962607; doi:10.1038/s41598-018-26223-9)
Supplement: Supplementary file 1 — Supplementary Information [file 41598_2018_26223_MOESM1_ESM.pdf]

Evaluation of weed control efficacy and crop safety of the new HPPD-inhibiting herbicide-QYR301

Hengzhi Wang[1,+], Weitang Liu[1,+], Kongping Zhao[1], Hui Yu[2], Jia Zhang[3], and Jinxin Wang[1,*]

*Correspondence should be addressed to J.W(email: wangjx@sdau.edu.cn)

[1] College of Plant Protection, Shandong Agricultural University, Tai'an, 271018, China.

[2] The Institute for the Control of Agrochemicals of Shandong Province, Ji'nan, 250013, China.

[3] The Institute for the Control of Agrichemicals of Ministry of Agriculture, Beijing, 100125, China.

[+] These authors contributed equally to this work.

Original data in greenhouse

Crop safety

Herbicide damage of the seedlings was visually estimated 21 d after treatment (DAT) using a scale of 0 to 100% (0 = no damage, 100 = total death)

three replicates and conducted twice.

soybean						
QYR301 180gai/ha	29	35	40	28	36	39
QYR301 360gai/ha	95	85	85	86	88	96
sunflower						
QYR301 180gai/ha	90	95	93	94	95	92
QYR301 360gai/ha	100	100	100	100	100	100
redbean						
QYR301 180gai/ha	25	30	40	25	40	33
QYR301 360gai/ha	70	70	75	75	70	75
radish						
QYR301 180gai/ha	100	98	99	100	99	100
QYR301 360gai/ha	98	100	98	100	100	100
greenbean						
QYR301 180gai/ha	20	15	15	18	15	23
QYR301 360gai/ha	45	44	60	48	50	55
wheat						
QYR301 180gai/ha	0	0	0	0	0	0
QYR301 360gai/ha	0	0	0	0	0	0
pepper						
QYR301 180gai/ha	20	30	30	25	30	28
QYR301 360gai/ha	45	46	55	45	48	47
peanut						
QYR301 180gai/ha	20	25	20	22	23	25
QYR301 360gai/ha	50	55	55	53	52	58
tomato						
QYR301 180gai/ha	75	70	60	65	62	68
QYR301 360gai/ha	85	95	95	90	92	93
carrot						
QYR301 180gai/ha	95	90	93	96	92	93
QYR301 360gai/ha	100	100	99	100	100	100
paddy						
QYR301 180gai/ha	0	0	0	0	0	0
QYR301 360gai/ha	0	0	0	0	0	0
greenonion						

QYR301 180gai/ha	73	60	75	75	72	68
QYR301 360gai/ha	91	88	92	95	98	97
eggplant						
QYR301 180gai/ha	85	75	68	72	69	79
QYR301 360gai/ha	95	82	89	87	85	92
garlic						
QYR301 180gai/ha	0	0	0	0	0	0
QYR301 360gai/ha	0	0	0	0	0	0
cotton						
QYR301 180gai/ha	15	14	20	18	20	15
QYR301 360gai/ha	50	59	64	55	52	53
watermelon						
QYR301 180gai/ha	33	34	35	33	33	35
QYR301 360gai/ha	73	74	77	75	75	74
cucumber						
QYR301 180gai/ha	52	54	53	52	53	58
QYR301 360gai/ha	77	72	84	77	82	75
potato						
QYR301 180gai/ha	32	40	45	38	35	35
QYR301 360gai/ha	72	60	73	72	71	69
corn						
QYR301 180gai/ha	0	0	0	0	0	0
QYR301 360gai/ha	0	0	0	0	0	0
rape						
QYR301 180gai/ha	56	70	59	60	62	65
QYR301 360gai/ha	95	90	97	95	92	95
cabbage						
QYR301 180gai/ha	65	77	72	68	70	72
QYR301 360gai/ha	72	85	88	77	82	85
sorghum						
QYR301 180gai/ha	15	17	29	18	12	28
QYR301 360gai/ha	35	29	31	30	31	33
caraway						
QYR301 180gai/ha	88	85	90	90	85	88
QYR301 360gai/ha	98	90	99	88	87	85

Weed spectrum

dry weight/pot 21DAT three replicates and conducted twice.

Echinochloa crusgali	1	2	3	1	2	3
CK	1.25	1.19	1.22	1.29	1.21	1.21
QYR301-60gai/ha	0.21	0.19	0.21	0.19	0.24	0.23
QYR301-120g ai/ha	0.18	0.15	0.16	0.13	0.15	0.16

Sclerochloa kengiana	1	2	3	1	2	3
CK	0.0811	0.0912	0.1011	0.0769	0.0777	0.0869
QYR301-60gai/ha	0.0899	0.0912	0.0887	0.0869	0.0899	0.0886
QYR301-120g ai/ha	0.0756	0.0756	0.0814	0.0779	0.0778	0.0812

Myosoton aquaticum	1	2	3	1	2	3
CK	0.3	0.38	0.55	0.44	0.32	0.48
QYR301-60gai/ha	0.15	0.15	0.16	0.18	0.17	0.18
QYR301-120g ai/ha	0.13	0.14	0.14	0.15	0.12	0.11

Polypogon fugax	1	2	3	1	2	3
CK	0.1523	0.2212	0.1799	0.2099	0.2313	0.1829
QYR301-60gai/ha	0.1099	0.1312	0.1088	0.1223	0.1022	0.1105
QYR301-120g ai/ha	0.0699	0.0612	0.0678	0.0601	0.0699	0.0811

Avena fatua	1	2	3	1	2	3
CK	0.54	0.52	0.49	0.51	0.52	0.51
QYR301-60gai/ha	0.45	0.51	0.48	0.48	0.5	0.49
QYR301-120g ai/ha	0.46	0.47	0.46	0.45	0.46	0.44

Alopecurus japonicus	1	2	3	1	2	3
CK	0.39	0.48	0.42	0.44	0.42	0.42
QYR301-60gai/ha	0.39	0.41	0.43	0.42	0.41	0.41
QYR301-120g ai/ha	0.39	0.39	0.4	0.41	0.39	0.38

Capsella bursa-pastoris	1	2	3	1	2	3
CK	0.1121	0.8417	0.1215	0.1236	0.0999	0.1236
QYR301-60gai/ha	0.0121	0.0074	0.0123	0.0089	0.0128	0.0089
QYR301-120g ai/ha	0.0189	0.0078	0.0069	0.0096	0.0089	0.0095

Galium aparine	1	2	3	1	2	3
CK	0.0712	0.0789	0.1212	0.0713	0.0689	0.0689
QYR301-60gai/ha	0.0789	0.0801	0.0789	0.0802	0.0769	0.0756
QYR301-120g ai/ha	0.0812	0.0756	0.0796	0.0756	0.0812	0.0799

Alopecurus aequalis	1	2	3	1	2	3
CK	0.15	0.2	0.2	0.18	0.21	0.22
QYR301-60gai/ha	0.17	0.2	0.19	0.21	0.22	0.19
QYR301-120g ai/ha	0.15	0.15	0.15	0.15	0.14	0.15

Beckmannia syzigachne	1	2	3	1	2	3
CK	0.33	0.34	0.31	0.35	0.32	0.31
QYR301-60gai/ha	0.35	0.36	0.34	0.35	0.36	0.37
QYR301-120g ai/ha	0.35	0.31	0.36	0.32	0.35	0.31

Geranium carolinianum	1	2	3	1	2	3
CK	0.2	0.16	0.15	0.18	0.19	0.2
QYR301-60gai/ha	0.12	0.12	0.13	0.13	0.12	0.12
QYR301-120g ai/ha	0.12	0.12	0.13	0.13	0.13	0.13

Pharbitis purpurea	1	2	3	1	2	3
CK	1.02	1.03	1.08	0.99	1.03	1.02
QYR301-60gai/ha	0.72	0.64	0.68	0.65	0.67	0.68
QYR301-120g ai/ha	0.54	0.53	0.58	0.58	0.54	0.57

Digitaria sanguinalis	1	2	3	1	2	3
CK	0.0712	0.0689	0.0712	0.0811	0.0712	0.0688
QYR301-60gai/ha	0.0756	0.0688	0.0708	0.0812	0.0699	0.0681
QYR301-120g ai/ha	0.0451	0.0398	0.0378	0.0461	0.0489	0.0478

Eleusine indica	1	2	3	1	2	3
CK	0.0588	0.0512	0.0632	0.0588	0.0578	0.0689
QYR301-60gai/ha	0.0203	0.0201	0.0209	0.0156	0.0199	0.0203

QYR301-120g ai/ha	0.0089	0.0091	0.0092	0.009	0.0091	0.0093
Cyperus rotundus	1	2	3	1	2	3
CK	0.0812	0.0802	0.0901	0.0812	0.0902	0.0799
QYR301-60gai/ha	0.0702	0.0699	0.0712	0.0703	0.0723	0.0707
QYR301-120g ai/ha	0.0614	0.0701	0.0708	0.0705	0.0704	0.0658
Scirpus juncoides	1	2	3	1	2	3
CK	0.0612	0.0545	0.0585	0.0587	0.0512	0.0574
QYR301-60gai/ha	0.0512	0.0509	0.0451	0.0489	0.0459	0.0489
QYR301-120g ai/ha	0.0412	0.0404	0.0359	0.0378	0.0412	0.0389
Setaria viridis	1	2	3	1	2	3
CK	0.1302	0.1256	0.1219	0.1389	0.1289	0.1302
QYR301-60gai/ha	0.0789	0.0796	0.0634	0.0702	0.0701	0.0699
QYR301-120g ai/ha	0.0512	0.0499	0.0601	0.0601	0.0523	0.0614
Echinochloa phyllopogon	1	2	3	1	2	3
CK	0.25	0.25	0.24	0.25	0.25	0.26
QYR301-60gai/ha	0.13	0.14	0.13	0.13	0.14	0.14
QYR301-120g ai/ha	0.0512	0.0502	0.0506	0.0503	0.0601	0.0513
Monochoria vaginalis	1	2	3	1	2	3
CK	0.0789	0.0896	0.0796	0.0912	0.0914	0.0896
QYR301-60gai/ha	0.0199	0.0156	0.0212	0.0189	0.0178	0.0175
QYR301-120g ai/ha	0.0085	0.0069	0.0078	0.0085	0.0086	0.0074
Abutilon theophrasti	1	2	3	1	2	3
CK	0.37	0.36	0.35	0.37	0.38	0.37
QYR301-60gai/ha	0.28	0.29	0.29	0.29	0.28	0.29
QYR301-120g ai/ha	0.21	0.21	0.2	0.2	0.22	0.21
Amaranthus retroflexus	1	2	3	1	2	3
CK	0.1612	0.1601	0.1376	0.1523	0.1589	0.1678
QYR301-60gai/ha	0.1689	0.1659	0.1689	0.1649	0.1656	0.1756

QYR301-120g ai/ha	0.0611	0.0601	0.0612	0.0612	0.0578	0.0589
Chenopodium serotinum	1	2	3	1	2	3
CK	0.1123	0.1201	0.1212	0.1236	0.1289	0.1159
QYR301-60gai/ha	0.0221	0.0211	0.0289	0.022	0.0256	0.0201
QYR301-120g ai/ha	0.0089	0.0056	0.0078	0.0056	0.0078	0.0065
Portulaca oleracea	1	2	3	1	2	3
CK	0.1629	0.1625	0.1689	0.1701	0.1645	0.1689
QYR301-60gai/ha	0.0889	0.1121	0.0989	0.0978	0.1002	0.102
QYR301-120g ai/ha	0.0912	0.0915	0.0936	0.0845	0.0912	0.0915
Eclipta prostrata	1	2	3	1	2	3
CK	0.1423	0.1442	0.1423	0.1507	0.1408	0.1478
QYR301-60gai/ha	0.0212	0.0289	0.0247	0.0318	0.0277	0.0287
QYR301-120g ai/ha	0.0078	0.0069	0.0071	0.0062	0.0078	0.0081
Solanum nigrum	1	2	3	1	2	3
CK	0.2456	0.0302	0.2789	0.3126	0.3201	0.2889
QYR301-60gai/ha	0.0412	0.0436	0.0469	0.0478	0.0459	0.0401
QYR301-120g ai/ha	0.0045	0.0012	0.0086	0.0074	0.0077	0.0055
Cyperus iria	1	2	3	1	2	3
CK	0.1121	0.1114	0.1145	0.1173	0.1107	0.1181
QYR301-60gai/ha	0.0076	0.0085	0.0085	0.0023	0.0056	0.0065
QYR301-120g ai/ha	0.0045	0.0056	0.0047	0.0061	0.0054	0.0048
Leptochloa chinensis	1	2	3	1	2	3
CK	0.1112	0.0865	0.1219	0.1355	0.1499	0.1199
QYR301-60gai/ha	0.0412	0.0389	0.0412	0.0385	0.0342	0.0451
QYR301-120g ai/ha	0.0089	0.0078	0.0086	0.0079	0.0083	0.0089
Bidens pilosa	1	2	3	1	2	3
CK	1.41	1.42	1.42	1.45	1.45	1.44
QYR301-60gai/ha	1.02	0.82	0.92	0.88	0.91	0.95
QYR301-120g ai/ha	0.71	0.62	0.72	0.58	0.72	0.59

Xanthium sibiricum	1	2	3	1	2	3
CK	1.38	1.21	1.33	1.35	1.41	1.21
QYR301-60gai/ha	1.08	1.12	1.21	1.12	1.21	1.08
QYR301-120g ai/ha	0.91	0.88	0.8	0.92	0.87	0.79

Heleocharis yokoscensis	1	2	3	1	2	3
CK	0.14	0.15	0.15	0.16	0.16	0.16
QYR301-60gai/ha	0.14	0.14	0.15	0.15	0.15	0.15
QYR301-120g ai/ha	0.15	0.16	0.14	0.15	0.17	0.15

Poa annua	1	2	3	1	2	3
CK	0.23	0.22	0.25	0.25	0.23	0.26
QYR301-60gai/ha	0.24	0.24	0.24	0.23	0.24	0.24
QYR301-120g ai/ha	0.25	0.24	0.24	0.23	0.24	0.23

Paddy hybrid tolerance.

Herbicide damage of the seedlings was visually estimated 21 d after treatment (DAT) using a scale of 0 to 100% (0 = no damage, 100 = total death)

	QYR301-180gai/ha			three replicates and conducted twice.			
Liangyouzao17	indica	22	25	22	28	19	25
zhongyou838	indica	22	18	22	15	22	17
liangyou287	indica	12	8	8	6	18	12
Dqibaoyou	indica	10	12	13	8	8	9
ganyuan100	indica	0	0	0	0	0	0
fengliangyou1	indica	0	0	0	0	0	0
rongyouhuazhan	indica	0	0	0	0	0	0
neixiang10	indica	0	0	0	0	0	0
Yliangyou1	indica	0	0	0	0	0	0
zhongjiazao17	indica	0	0	0	0	0	0
huanghuazhan	indica	0	0	0	0	0	0
huajing5	japonica	0	0	0	0	0	0
huaidao5	japonica	0	0	0	0	0	0
lianjing11	japonica	0	0	0	0	0	0
longjing39	japonica	0	0	0	0	0	0
longjing46	japonica	0	0	0	0	0	0
nanjing9108	japonica	0	0	0	0	0	0
ningjing28	japonica	0	0	0	0	0	0

ningjing33	japonica	0	0	0	0	0	0
ningjing45	japonica	0	0	0	0	0	0
shengdao16	japonica	0	0	0	0	0	0
tongjing89	japonica	0	0	0	0	0	0
xuhuan168	japonica	0	0	0	0	0	0
yanfeng47	japonica	0	0	0	0	0	0
zhendao99	japonica	0	0	0	0	0	0
yujing6	japonica	0	0	0	0	0	0
fuyuan4	japonica	0	0	0	0	0	0
longjing47	japonica	0	0	0	0	0	0
jinxiangnuo	indica	0	0	0	0	0	0
	QYR301-360gai/ha						
Liangyouzao17	indica	52	56	55	65	58	59
zhongyou838	indica	45	42	44	41	41	42
liangyou287	indica	52	59	51	48	53	55
Dqibaoyou	indica	15	13	18	12	15	12
ganyuan100	indica	22	22	21	15	18	17
fengliangyou1	indica	15	12	15	12	16	18
rongyouhuazhan	indica	12	15	18	12	15	15
neixiang10	indica	12	11	8	9	15	12
Yliangyou1	indica	5	5	6	5	6	4
zhongjiazao17	indica	5	6	8	8	8	5
huanghuazhan	indica	0	0	0	0	0	0
huajing5	japonica	0	0	0	0	0	0
huaidao5	japonica	0	0	0	0	0	0
lianjing11	japonica	0	0	0	0	0	0
longjing39	japonica	0	0	0	0	0	0
longjing46	japonica	0	0	0	0	0	0
nanjing9108	japonica	0	0	0	0	0	0
ningjing28	japonica	0	0	0	0	0	0
ningjing33	japonica	0	0	0	0	0	0
ningjing45	japonica	0	0	0	0	0	0
shengdao16	japonica	0	0	0	0	0	0
tongjing89	japonica	0	0	0	0	0	0
xuhuan168	japonica	0	0	0	0	0	0
yanfeng47	japonica	0	0	0	0	0	0
zhendao99	japonica	0	0	0	0	0	0
yujing6	japonica	7	6	10	3	3	2

fuyuan4	japonica	0	0	0	0	0	0		
longjing47	japonica	0	0	0	0	0	0		
jinxiangnuo	indica	0	0	0	0	0	0		
	Selectivity index (SI)								
latewatergrass		dryweight(g)/pot 21DAT			three replicates and conducted twice.				
CK		0.3126	0.2889	0.3215			0.3312	0.3129	0.3245
11.25	gai/ha	0.2987	0.2886	0.2912			0.3012	0.2866	0.2765
22.5	gai/ha	0.2445	0.2813	0.2588			0.2536	0.2612	0.2588
45	gai/ha	0.1699	0.1389	0.1512			0.1601	0.1545	0.1532
90	gai/ha	0.102	0.1078	0.1232			0.0789	0.0912	0.0932
180	gai/ha	0.0489	0.0402	0.0312			0.03611	0.0378	0.0362
360	gai/ha	0.0312	0.0215	0.0356			0.0299	0.0312	0.0322
baranyardgrass									
CK		1.42	1.29	1.5			1.42	1.55	1.35
3.75	gai/ha	1.11	1.39	1.25			1.28	1.29	1.31
7.5	gai/ha	0.96	1.16	1.2			1.13	1.17	1.23
15	gai/ha	0.96	0.89	0.95			0.82	1.03	0.91
30	gai/ha	0.56	0.48	0.69			0.57	0.57	0.55
60	gai/ha	0.25	0.27	0.27			0.25	0.26	0.23
120	gai/ha	0.15	0.13	0.14			0.11	0.10	0.12
shepherd's purse									
CK		0.2614	0.2745	0.2239			0.2479	0.2412	0.2719
1.875	gai/ha	0.1889	0.2178	0.2212			0.2036	0.2069	0.2117
3.75	gai/ha	0.1778	0.1646	0.1975			0.1812	0.1812	0.1793
7.5	gai/ha	0.1021	0.1331	0.1289			0.1345	0.1096	0.1231
15	gai/ha	0.0588	0.1001	0.0793			0.0812	0.0836	0.0802
30	gai/ha	0.0532	0.0245	0.0412			0.0289	0.0521	0.0399
60	gai/ha	0.0212	0.0189	0.0076			0.0089	0.0075	0.0069
sheathed monochoria herb									
CK		0.0789	0.0896	0.0796			0.0912	0.0914	0.0896
3.75	gai/ha	0.0812	0.0819	0.0814			0.0789	0.0796	0.0823
7.5	gai/ha	0.0736	0.0789	0.0785			0.0741	0.0736	0.0726
15	gai/ha	0.0536	0.0536	0.0578			0.0576	0.0529	0.0589
30	gai/ha	0.0396	0.0412	0.0312			0.0402	0.0369	0.0411

60	gai/ha	0.0199	0.0156	0.0212	0.0189	0.0178	0.0175
120	gai/ha	0.0085	0.0069	0.0078	0.0085	0.0086	0.0074
black nightshade							
CK		0.2412	0.2745	0.2536	0.2103	0.2874	0.2645
0.1172	gai/ha	0.2212	0.2315	0.2214	0.2311	0.2015	0.2102
0.4686	gai/ha	0.1896	0.1823	0.1945	0.1956	0.1845	0.1956
1.875	gai/ha	0.1102	0.0987	0.1236	0.1145	0.1234	0.1245
7.5	gai/ha	0.0725	0.0715	0.0712	0.0687	0.0612	0.0645
30	gai/ha	0.0256	0.0289	0.0278	0.0301	0.0296	0.0312
120	gai/ha	0.0089	0.0096	0.0095	0.0085	0.0096	0.0094
rice flat sedge							
CK		0.2478	0.2812	0.2732	0.2632	0.2589	0.2612
3.75	gai/ha	0.2125	0.2145	0.2178	0.2201	0.2156	0.2147
7.5	gai/ha	0.2021	0.2101	0.1889	0.1781	0.1821	0.1938
15	gai/ha	0.1416	0.1521	0.1611	0.1345	0.1421	0.1219
30	gai/ha	0.1189	0.0709	0.1114	0.0981	0.1012	0.0912
60	gai/ha	0.0631	0.0507	0.0612	0.0589	0.0602	0.0564
120	gai/ha	0.0286	0.0211	0.0302	0.0256	0.0245	0.0311
chinese sprangletop							
CK		0.1193	0.1285	0.1196	0.1321	0.1241	0.1312
3.75	gai/ha	0.1023	0.1085	0.0992	0.1121	0.1088	0.1031
7.5	gai/ha	0.0901	0.0963	0.0915	0.0911	0.1056	0.0856
15	gai/ha	0.0689	0.0712	0.0756	0.0752	0.0739	0.0812
30	gai/ha	0.0512	0.0536	0.0425	0.0456	0.0521	0.0489
60	gai/ha	0.0201	0.0269	0.0311	0.0156	0.0278	0.0212
120	gai/ha	0.0036	0.0045	0.0051	0.0051	0.0021	0.0039
Eclipta prostrata							
CK		1.0214	0.8825	0.9911	1.0200	1.1121	0.9612
3.75	gai/ha	0.8612	0.8814	0.9125	0.8512	0.8312	0.9212
7.5	gai/ha	0.7121	0.6785	0.8512	0.7125	0.7202	0.8612
15	gai/ha	0.6611	0.7114	0.7721	0.6200	0.6211	0.5889
30	gai/ha	0.4913	0.4632	0.4412	0.4236	0.4512	0.4469
60	gai/ha	0.1631	0.1436	0.1785	0.1723	0.1624	0.1616

120	gai/ha	0.0511	0.0769	0.0689	0.0526	0.0511	0.0623
jining 28	rice						
CK		1.9612	1.8879	1.9623	1.7936	1.8925	2.0912
540	gai/ha	1.7912	1.7623	1.8369	1.9312	1.9612	1.6216
1080	gai/ha	1.6912	1.7536	1.8236	1.7125	1.7716	1.7812
1440	gai/ha	1.5633	1.4926	1.5126	1.5326	1.4547	1.4836
2160	gai/ha	0.7412	0.8912	0.6936	0.7124	0.6825	0.6639
4320	gai/ha	0.2312	0.2136	0.1819	0.2545	0.2178	0.1996
8640	gai/ha	0.1525	0.1326	0.1445	0.1526	0.1723	0.1364
zhongjiazao17	rice						
CK		0.6618	0.6728	0.6876	0.7121	0.6916	0.6548
360	gai/ha	0.6512	0.6421	0.6615	0.6812	0.6512	0.6636
540	gai/ha	0.6212	0.6012	0.6512	0.6514	0.6319	0.6618
720	gai/ha	0.5512	0.5812	0.5612	0.5912	0.5912	0.5745
1080	gai/ha	0.3362	0.3615	0.3718	0.3618	0.4121	0.2912
1440	gai/ha	0.1215	0.1291	0.2105	0.1819	0.2106	0.2206
2160	gai/ha	0.1102	0.0998	0.1012	0.1512	0.1032	0.1135

Activity of QYR301 against resistant weeds in paddy.

	dry weight/pot 21DAT			three replicates and conducted twice.		
latewatergrass-ALS-R	1	2	3	1	2	3
CK	0.7980	0.7700	0.7219	0.8125	0.7635	0.8029
Penoxsulam	0.7921	0.7167	0.8695	0.8714	0.8042	0.8012
QYR301 120g ai/ha	0.2914	0.2533	0.2550	0.2122	0.2345	0.2618
QYR301 180g ai/ha	0.0563	0.0500	0.0530	0.0446	0.0678	0.0579
latewatergrass-S						
CK	1.4808	1.8718	1.8045	1.5876	1.7512	1.6325
Penoxsulam 15gai/ha	0.5288	0.5321	0.5064	0.4612	0.5121	0.4736
cyhalofop-butyl 80gai/ha	0.2628	0.3942	0.3269	0.2136	0.1928	0.2312
QYR301 120g ai/ha	0.3590	0.5096	0.3269	0.4121	0.3321	0.2989
QYR301 180g ai/ha	0.0673	0.0673	0.0801	0.0512	0.0932	0.1021

latewatergrass-ACCase-R						
CK	1.1172	1.0780	1.0106	1.1375	1.0689	1.1241
cyhalofop-butyl 80gai/ha	0.9921	0.9893	1.0849	1.0097	0.9785	0.9643
QYR301 120g ai/ha	0.3264	0.2837	0.2856	0.2377	0.2626	0.2932
QYR301 180g ai/ha	0.0630	0.0560	0.0593	0.0500	0.0759	0.0648
baranyardgrass-S						
CK	0.9547	0.8285	0.9320	1.0236	1.1121	0.9636
Quinclorac 250g ai/ha	0.1068	0.0518	0.0547	0.0714	0.0812	0.1001
QYR301 60gai/ha	0.0809	0.1003	0.0712	0.0799	0.0812	0.0936
QYR301 120gai/ha	0.0615	0.0712	0.0680	0.0811	0.0785	0.1001
Penoxsulam 15gai/ha	0.1012	0.0698	0.0812	0.0745	0.0867	0.0666
barnyardgrass-Q-R						
CK	0.9803	1.0230	0.9344	1.1236	0.9876	1.0879
Quinclorac 250g ai/ha	0.6869	0.6712	0.6230	0.6975	0.7215	0.7512
QYR301 60gai/ha	0.0754	0.0820	0.1344	0.0876	0.0912	0.0769
QYR301 120gai/ha	0.0721	0.0557	0.0852	0.0812	0.0925	0.0774
barnyardgrass-ALS-R						
CK	0.9803	1.0230	0.9344	1.1236	0.9876	1.0879
Penoxsulam 15gai/ha	1.1215	0.9546	1.2136	1.0021	1.2136	0.9989
QYR301 60gai/ha	0.1261	0.0912	0.0845	0.0912	0.0902	0.0889
QYR301 120gai/ha	0.0966	0.0935	0.1236	0.1512	0.0912	0.0874

Qriginal data in field experiments

	**crop safety**				
**Ji'ning**	Paddy hybrid:tiyou267	crop injury 3 day after treatment （%）			
		1	2	3	4
1	QYR301 90 g ai/ha	0	0	0	0
2	QYR301 135 g ai/ha	0	0	0	0
3	QYR301 180 g ai/ha	0	0	0	0
4	QYR301 270 g ai/ha	3	6	2	5
5	Penoxsulam 30g ai/ha	0	0	0	0
6	weed control				
7	hand weeding				
		crop injury 5 day after treatment （%）			
		1	2	3	4
1	QYR301 90 g ai/ha	0	0	0	0
2	QYR301 135 g ai/ha	0	0	0	0
3	QYR301 180 g ai/ha	0	0	0	0
4	QYR301 270 g ai/ha	7	8	3	5
5	Penoxsulam 30g ai/ha	0	0	0	0
6	weed control				
7	hand weeding				
		crop injury 10 day after treatment （%）			
		1	2	3	4
1	QYR301 90 g ai/ha	0	0	0	0
2	QYR301 135 g ai/ha	0	0	0	0
3	QYR301 180 g ai/ha	0	0	0	0
4	QYR301 270 g ai/ha	2	2	1	1
5	Penoxsulam 30g ai/ha	0	0	0	0
6	weed control				
7	hand weeding				
		crop injury 20 day after treatment （%）			
		1	2	3	4
1	QYR301 90 g ai/ha	0	0	0	0

2	QYR301 135 g ai/ha	0	0	0	0
3	QYR301 180 g ai/ha	0	0	0	0
4	QYR301 270 g ai/ha	0	0	0	0
5	Penoxsulam 30g ai/ha	0	0	0	0
6	weed control				
7	hand weeding				

**shi'zui'shan**	Paddy hybrids:ningjing48	crop injury 3 day after treatment （%）			
		1	2	3	4
1	QYR301 90 g ai/ha	0	0	0	0
2	QYR301 135 g ai/ha	0	0	0	0
3	QYR301 180 g ai/ha	0	0	0	0
4	QYR301 270 g ai/ha	0	0	0	0
5	Penoxsulam 30g ai/ha	0	0	0	0
6	weed control				
7	hand weeding				

		crop injury 5 day after treatment （%）			
		1	2	3	4
1	QYR301 90 g ai/ha	0	0	0	0
2	QYR301 135 g ai/ha	0	0	0	0
3	QYR301 180 g ai/ha	0	0	0	0
4	QYR301 270 g ai/ha	0	0	0	0
5	Penoxsulam 30g ai/ha	0	0	0	0
6	weed control				
7	hand weeding				

		crop injury 10 day after treatment （%）			
		1	2	3	4
1	QYR301 90 g ai/ha	0	0	0	0
2	QYR301 135 g ai/ha	0	0	0	0
3	QYR301 180 g ai/ha	0	0	0	0
4	QYR301 270 g ai/ha	0	0	0	0
5	Penoxsulam 30g ai/ha	0	0	0	0

6	weed control				
7	hand weeding				

		crop injury 20 day after treatment（%）			
		1	2	3	4
1	QYR301 90 g ai/ha	0	0	0	0
2	QYR301 135 g ai/ha	0	0	0	0
3	QYR301 180 g ai/ha	0	0	0	0
4	QYR301 270 g ai/ha	0	0	0	0
5	Penoxsulam 30g ai/ha	0	0	0	0
6	weed control				
7	hand weeding				

Weed control efficacy

**Ji ‘ning**		efficacy 20days after treatments							
		barnyard grass					Chinese sprangletop		
		1	2	3	4	1	2	3	4
1	QYR301 90 g ai/ha	89	92	91	88	53	52	56	58
2	QYR301 135 g ai/ha	95	94	94	95	89	88	85	92
3	QYR301 180 g ai/ha	99	98	98	96	95	94	95	96
4	QYR301 270 g ai/ha	99	98	99	96	98	99	99	97
5	Penoxsulam 30g ai/ha	95	99	100	96	32	33	35	38
6	weed control								
7	hand weeding								
		efficacy 40 days after treatments（%）							
		barnyard grass					Chinese sprangletop		
		1	2	3	4	1	2	3	4
1	QYR301 90 g ai/ha	84	83	84	86	52	43	45	49
2	QYR301 135 g ai/ha	93	92	92	94	85	84	86	88
3	QYR301 180 g ai/ha	95	96	98	95	94	93	95	93
4	QYR301 270 g ai/ha	99	98	96	97	96	98	99	95
5	Penoxsulam 30g ai/ha	94	95	98	98	32	23	35	32
6	weed control								
7	hand weeding								

shi'zuishan

efficacy 20days after treatments（%）

		barnyard grass					Chinese sprangletop		
		1	2	3	4	1	2	3	4
1	QYR301 90 g ai/ha	88	89	87	88	49	49	52	53
2	QYR301 135 g ai/ha	94	93	94	94	83	86	88	87
3	QYR301 180 g ai/ha	97	96	97	95	94	93	93	94
4	QYR301 270 g ai/ha	99	99	98	99	97	97	99	99
5	Penoxsulam 30g ai/ha	35	32	33	35	28	26	23	24
6	weed control								
7	hand weeding								

efficacy 40 days after treatments（%）

		barnyard grass					Chinese sprangletop		
		1	2	3	4	1	2	3	4
1	QYR301 90 g ai/ha	82	85	85	83	47	48	45	48
2	QYR301 135 g ai/ha	94	93	92	92	84	82	85	85
3	QYR301 180 g ai/ha	97	96	98	96	93	92	92	93
4	QYR301 270 g ai/ha	99	98	97	97	98	95	96	98
5	Penoxsulam 30g ai/ha	32	33	35	31	23	25	28	23
6	weed control								
7	hand weeding								

rice yield

Ji'ning Paddy hybrid:Tiyou267 yield/5m2 (kg)

			1	2	3	4
1	QYR301	90 g ai/ha	2.03	2.11	2.13	2.31
2	QYR302	135g ai/ha	2.21	2.29	2.31	2.34
3	QYR303	180g ai/ha	2.33	2.35	2.33	2.41
4	QYR304	270g ai/ha	2.32	2.36	2.39	2.47
5	Penoxsulam	30g ai/ha	2.31	2.27	2.21	2.26
6	Hand weeding		1.96	2.01	2.12	1.98
7	weed control		2.38	2.39	2.45	2.49

Shi'zui'shan Paddy hybrids:Ningjing48 yield/5m2 (kg)

			1	2	3	4
1	QYR301	90 g ai/ha	5.33	5.47	5.45	5.41

2	QYR302	135g ai/ha	5.55	5.71	5.61	5.45
3	QYR303	180g ai/ha	5.96	5.89	5.98	5.81
4	QYR304	270g ai/ha	6.02	6.05	5.99	5.79
5	Penoxsulam	30g ai/ha	5.15	4.99	5.15	5.23
6	Hand weeding		5.12	5.12	5.01	5.21
7	weed control		6.11	6.12	6.09	5.99
